# Prosthetic rehabilitation of a patient with X-linked hypophosphatemia using dental implants: a case report and review of the literature

**DOI:** 10.1186/s40729-019-0169-3

**Published:** 2019-04-22

**Authors:** Martin James, Reza Vahid Roudsari

**Affiliations:** 1 Department of Restorative Dentistry, University Dental Hospital of Manchester, Manchester University NHS Foundation Trust, Higher Cambridge Street, Manchester, M15 6FH UK; 2 School of Medical Sciences, Faculty of Biology, Medicine and Health, The University of Manchester, Oxford Road, Manchester, M13 9PL UK

**Keywords:** X-linked hypophosphatemia, Dental implant, Cement-retained restoration, Screw-retained restoration, Connective tissue graft, Block bone graft, Xenograft

## Abstract

**Background:**

X-linked hypophosphatemia is associated with a range of dental problems, many of which may result in early loss of the dentition. Most patients, but especially young adults, are likely to desire fixed prosthodontic replacements, and dental implants may be the preferred solution in many cases. The use of dental implants to rehabilitate this patient group has not been widely studied with the literature limited to a small number of case reports with limited follow-up.

**Case presentation:**

This case report describes the dental journey of a young adult with X-linked hypophosphatemia, his eventual tooth loss and rehabilitation with multiple dental implants. Over 10 years’ follow-up of three of the fixtures is presented.

**Conclusions:**

This case report shows a common presentation and progression of a patient with X-linked hypophosphatemia and will hopefully provide further positive evidence for the clinician to rely on when considering dental implant based treatments for such patients.

## Background

X-linked hypophosphatemia (XLH) was first described by Albright [1] as Vitamin D-resistant rickets and is the most common hereditary metabolic rickets with a prevalence of 1:20,000 [2]. The genetic mutation is of the phosphate-regulating gene PHEX which results in reduced phosphate reabsorption by the kidneys [3] and due to the dependency between calcium and phosphate homeostasis results in bone deformities (particularly bowing of the lower extremities), bone pain, reduced growth, hypophosphatemia, inappropriately normal serum 1,25(OH)2D levels and phosphate wasting [4]. There is a general lack of information about XLH and treatment guidelines which frequently lead to missed diagnoses and mismanagement [5].

The dental implications of XLH manifest clinically as recurrent abscess and sinus tract formation associated with non-carious teeth [6–19] as well as delayed tooth eruption [12, 19] in both the primary and in the permanent dentitions. Edentulous regions generally have hypoplastic alveolar ridges [17, 20]. Radiographic appearance is classically of large pulp chambers [6, 8, 10–14, 17–23] with a high pulp-to-tooth volume ratio (taurodontism) [6, 8, 9, 12, 16]. A thin enamel layer [6, 8, 10, 18, 20, 21, 23] along with dentinal defects [6, 8, 18, 20, 23, 24], short roots [11, 20, 23], root resorptions in the primary dentition [16, 20, 22], a poorly defined lamina dura [10, 16, 17, 20] and an increase in the prevalence and severity of periodontal disease [25] are also seen.

Histologically, the dentine is characterised by large tubular clefts or lacunae [9, 17, 23] with prominent pulp horns [7, 8, 10, 13, 16, 17, 19, 21, 22, 26] often extending up to and beyond the amelo-dentinal junction (ADJ) [6–10, 13, 16, 17, 19, 21, 22] particularly in the primary teeth [8, 10, 11, 19, 22, 27–29]. The dentine layer is usually thin [8, 30] and with areas of unmineralised dentine [6, 10, 16, 18, 21, 23, 30–32], a wide predentine zone and tubular defects [6, 10, 13, 23, 24]. This appearance is due to a lack of fusion of calcospherites and consequently the presence of large interglobular spaces [7, 22, 24]. There is also a lack of secondary dentine formation [33].

There is an acellular cementum hypoplasia especially in patients with late or incomplete supplement treatment [25]. The enamel is hypoplastic and presents with cracks extending from the surface to the ADJ [9, 10, 12, 14, 18, 21, 23, 34]. It is these enamel cracks along with the dentinal defects described which allow microbial penetration into the pulp chamber resulting in the clinical presentation seen [22].

Older patients with XLH have more experience of dental abscesses [26], and the most frequently affected teeth are incisors and canines followed by the molars [26]. This pattern of presentation is thought to be due to both the sequence of eruption and also the rate of attrition exposing defective dentine to microbial invasion [10, 17, 24].

Medical treatment for XLH usually consists of oral phosphate supplements and calcitriol. This aims to reduce the hypophosphatemia but prevents the development of secondary hyperparathyroidism [35]. Systemic treatment has been shown to prevent or treat dental anomalies by some authors [8, 19, 25, 36], but others have found little dental benefit [8, 14, 37, 38].

Due to the wide variety of dental presentations, there is no consensus on the most appropriate treatment modality [39]. The most commonly agreed upon recommendations are regular dental review including sensitivity testing of all teeth and radiographic examination of the entire dentition [34].

There is a dearth of literature on the use of dental implants in patients diagnosed with XLH consisting of a handful of case reports with short follow-up. Considering the likelihood that young patients with multiple missing teeth and rapidly failing dentitions will desire fixed rehabilitations, implant-supported prosthesis may be the preferred treatment modality. The aim of this paper is to provide long term evidence that implant-supported prostheses can be successful in this patient group and give the clinician considering such an approach confidence by reporting a case of a young patient with XLH who has been rehabilitated with four dental implant-supported restorations, utilising five fixtures, and a follow-up of 1 year 9 months to 10 years 3 months.

## Case report

**Fig. 1:**
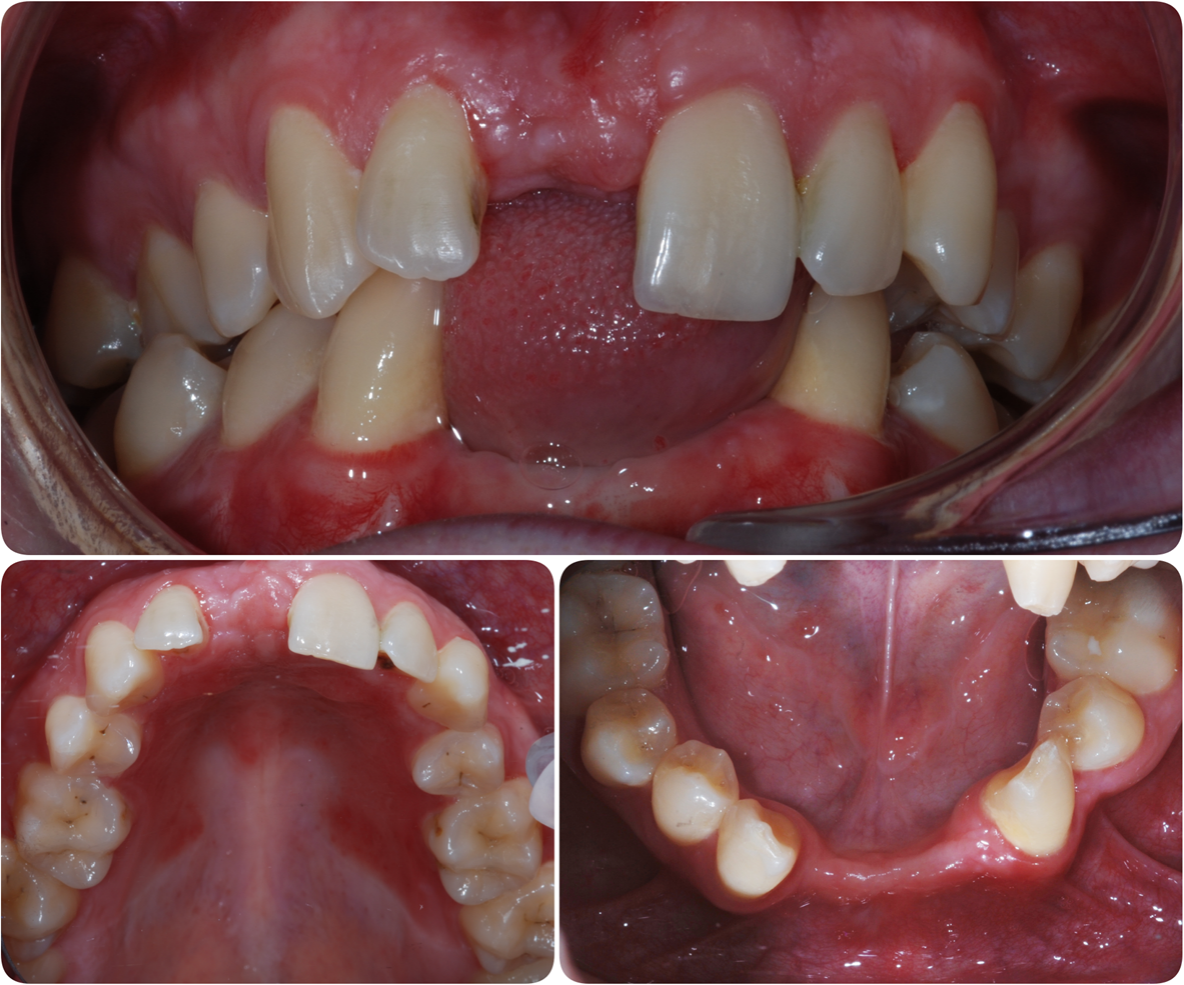
Pre-operative photographs. Clinical situation at presentation

**Fig. 2:**
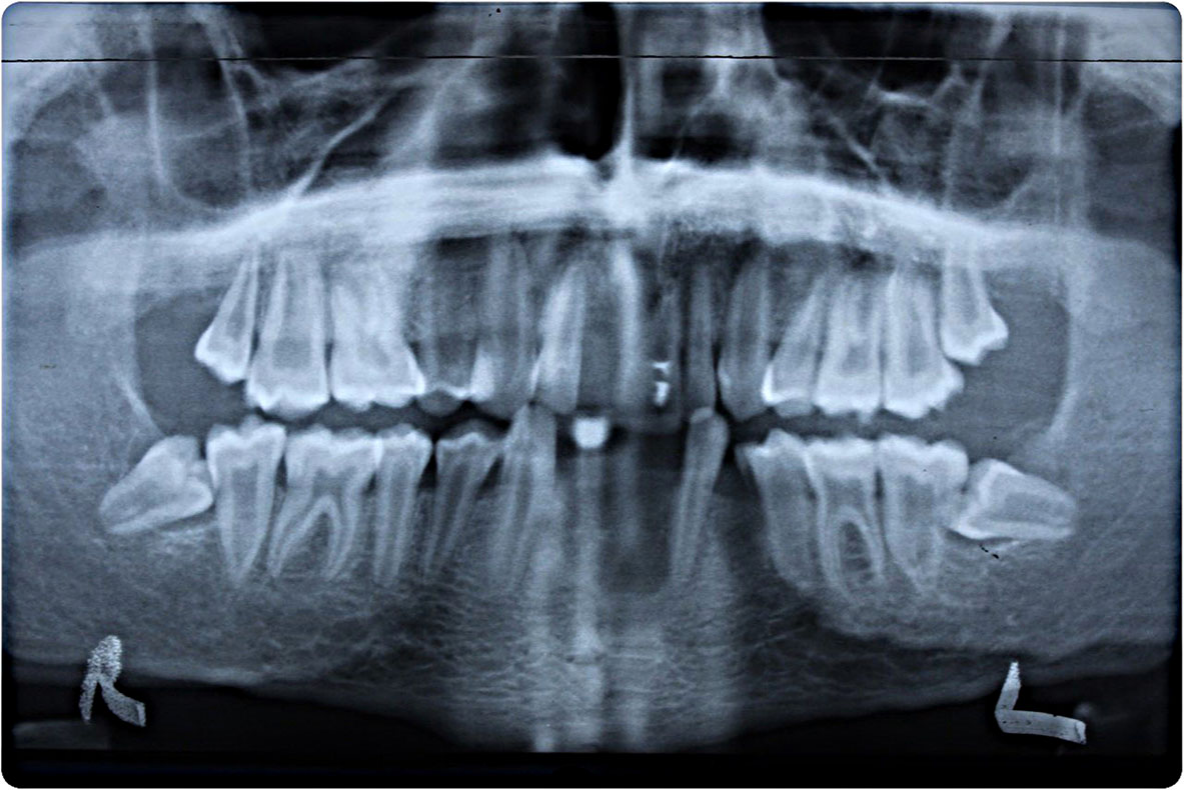
Pre-operative radiographs. Radiographic situation at presentation

In March 2006, an 18-year-old male attended the Department of Restorative Dentistry at The University Dental Hospital of Manchester. He was missing teeth 11, 31, 32, 41 and 42 (Figs. 1 and 2) and was wearing partial acrylic prostheses; he was otherwise fully dentate and disease free.

Medically, the patient, along with his sister, had been diagnosed with XLH at the age of 6 months. He had been taking oral phosphate supplements and calcitriol since childhood and had also undergone multiple surgical procedures to correct bowing of his legs. He was a non-smoker.

The patient had a complex dental history which began in The Department of Paediatric Dentistry at The University Dental Hospital of Manchester at the age of 1 year and culminated in his care being transferred to The Department of Restorative Dentistry on his 16th birthday. During this time, he had undergone the extraction of many his primary dentition due to spontaneous pulpal necrosis, periapical pathology and abscess formation, and he also had teeth 11, 21, 31, 32, 41 and 42 extirpated and dressed with non-setting calcium hydroxide for the same reasons in April–May 2003. Between June 2004 and June 2005, teeth 11, 31, 32, 41 and 42 all fractured unrestorably at gingival level and so were extracted in August 2005 and partial acrylic prostheses provided.

**Fig. 3:**
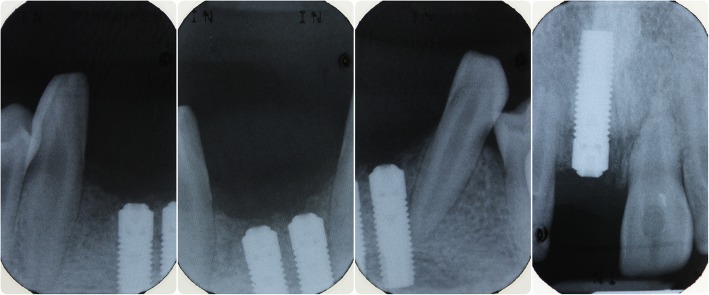
Placement confirmation. Radiograph of fixtures in 11, 31 and 41 positions confirming angulation

**Fig. 4:**
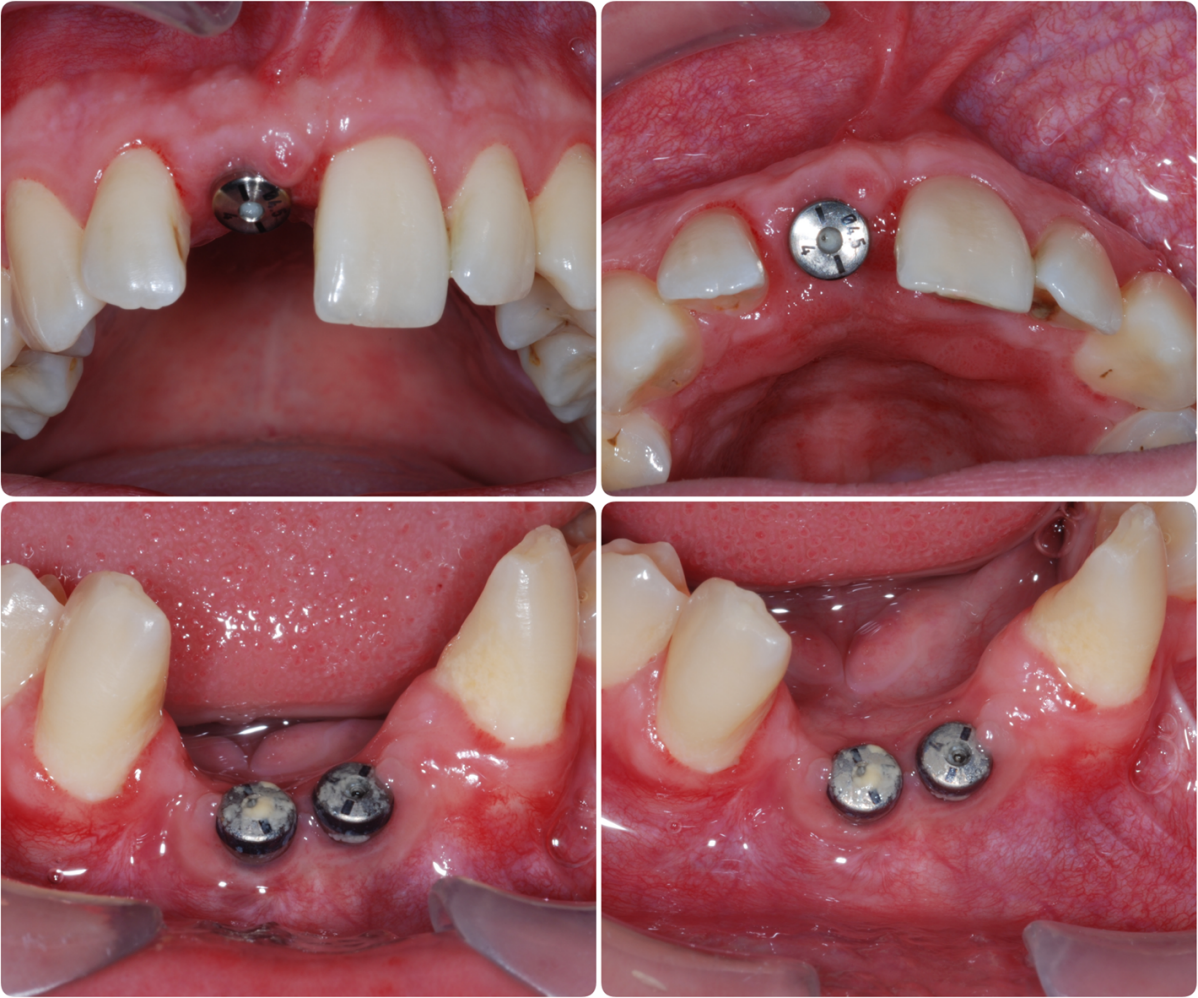
Healing following fixture exposure. Fixtures following secondary surgery and connection of healing abutments in 11, 31 and 41 positions

**Fig. 5:**
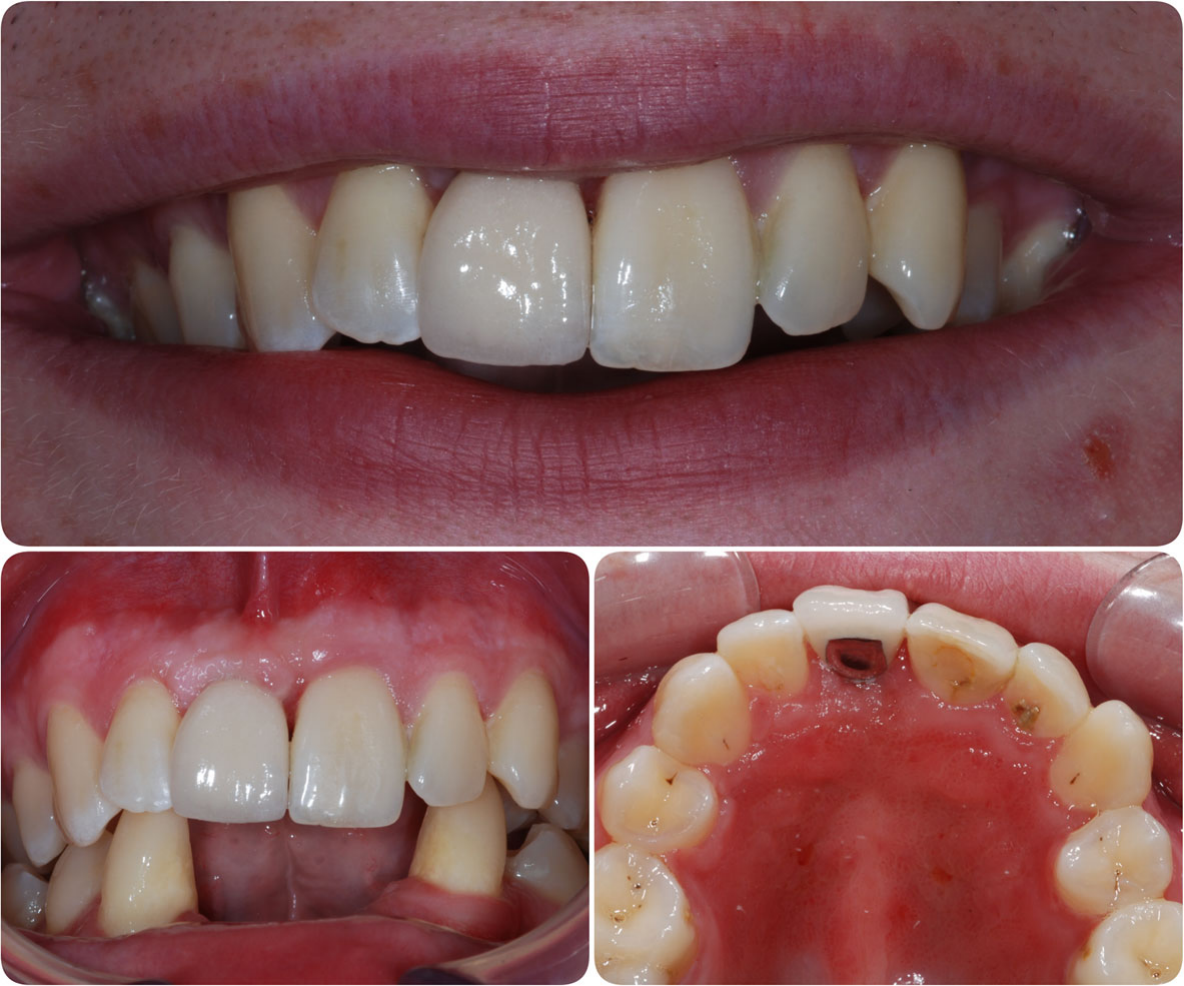
Maxilla restored. Restored fixture in 11 position, 31 and 41 position fixtures remains unrestored as awaiting a connective tissue graft due to a narrow band of attached gingivae labial to the fixtures

In May 2007, a block graft harvested from the chin was used to augment the lower anterior region, and following 6 months of healing, three fixtures (Astra OsseoSpeed©, Dentsply Implants, Mölndal, Sweden) were placed; 4.0-mm diameter and 15-mm length in the 11 position and 13-mm length in the 31 and 41 positions (Fig. 3). Following 3 months for osseointegration, all implants were exposed in a second procedure (Fig. 4); however, due to the patient having a very narrow band of keratinised mucosa anterior to the implants in the 31 and 41 positions, which was painful to brush and therefore leading to poor oral hygiene in this area, a connective tissue graft was desired. Unfortunately, the patient had developed denture stomatitis of the palate, and this could not be resolved with hygiene instruction and topical antifungals, so, in May 2008, it was decided to restore the implant in the 11 position with a cement-retained porcelain-fused-to-metal (PFM) restoration (Fig. 5), allowing the patient to stop wearing his upper prosthesis and resolution of the stomatitis.

**Fig. 6:**
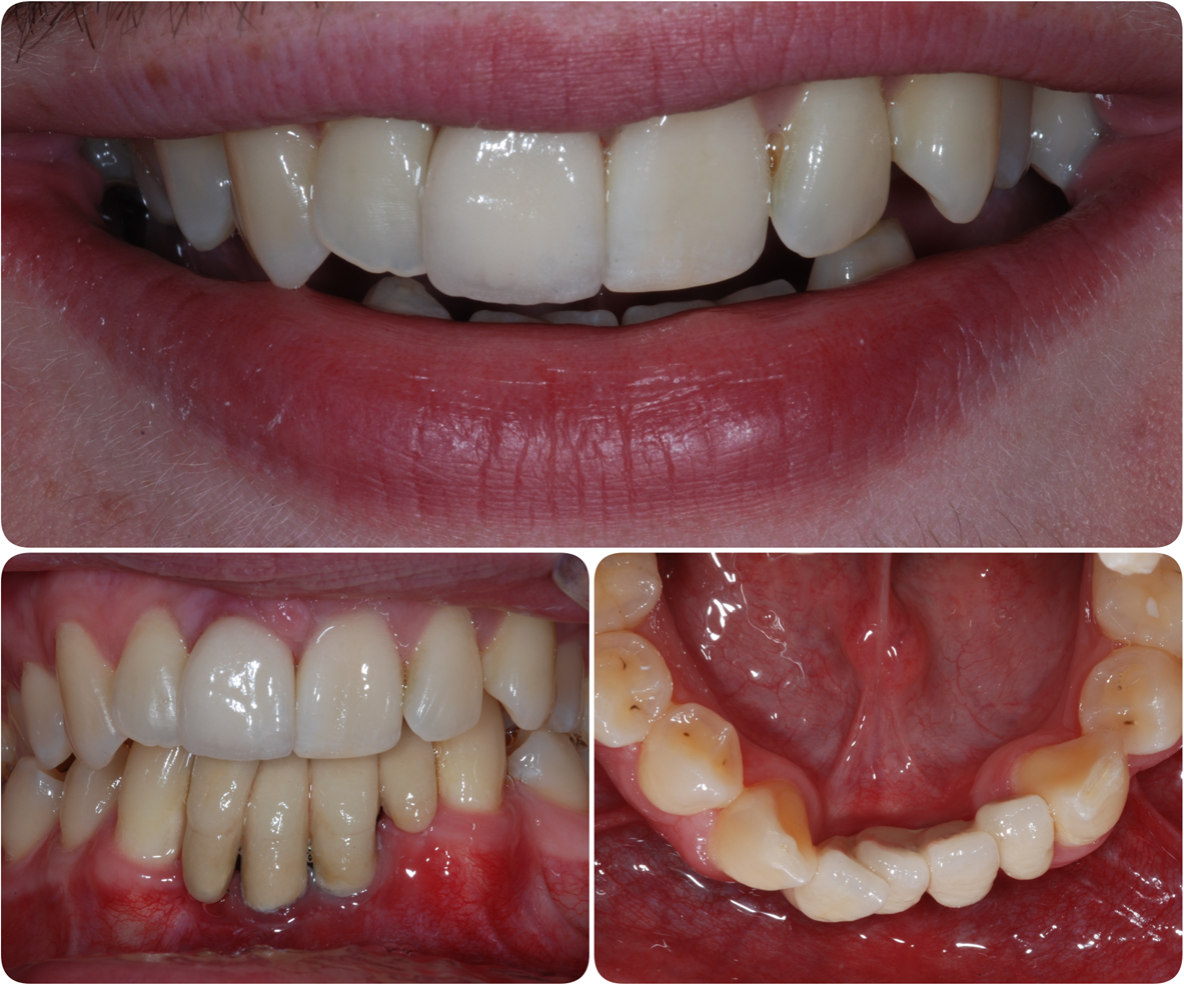
Mandible restored. Fixtures in 31 and 41 positions restored with four-unit bridge

In January 2009, a connective tissue graft was harvested from the palate and used to provide an increased band of attached gingivae buccal to the lower anterior fixtures which were then, 5 months later, restored with a four-unit cement-retained PFM bridge with two single-unit distal cantilevers (Fig. 6).

**Fig. 7:**
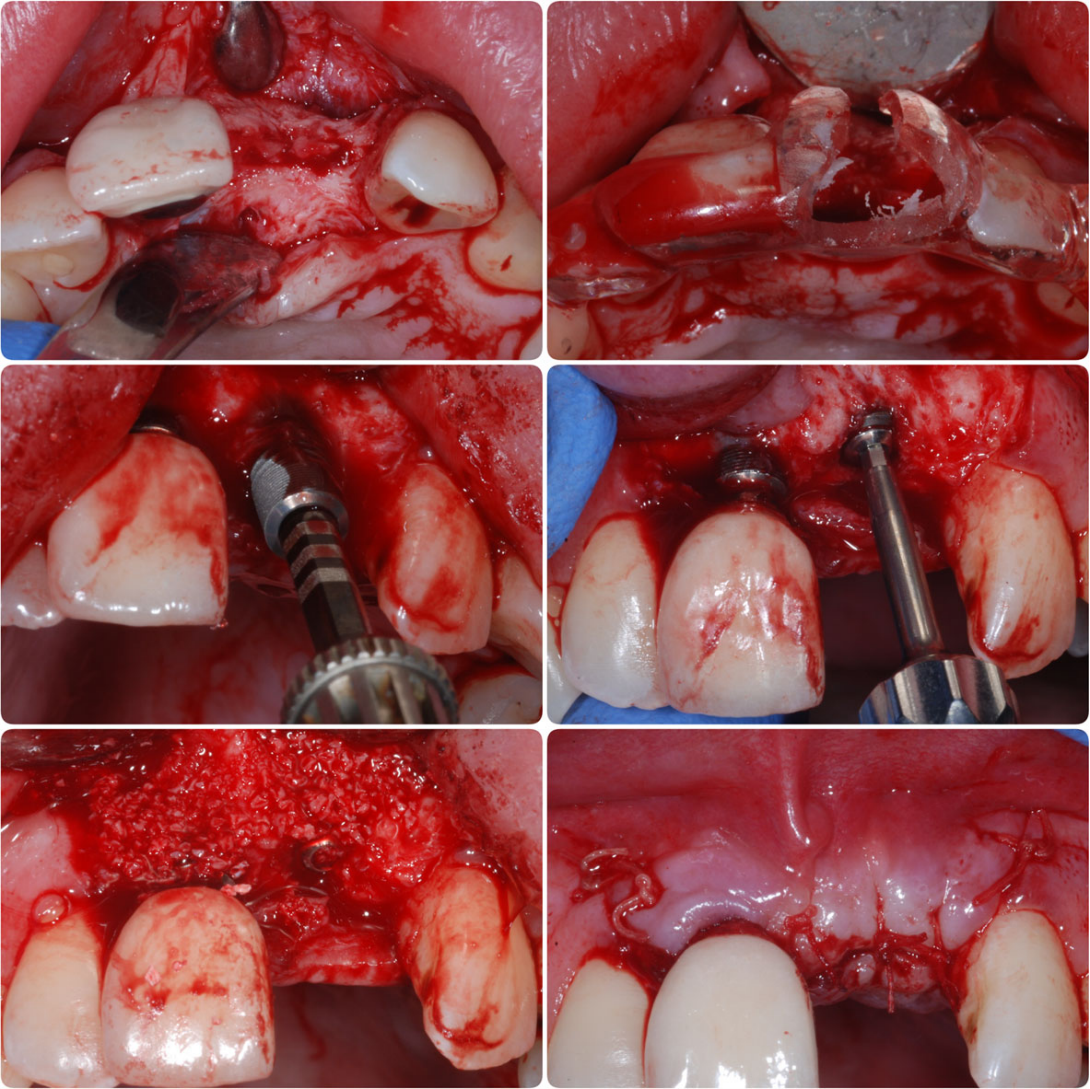
Additional fixture placement. Surgical stages in fixture placement in position 21 and simultaneous particulate bone graft

**Fig. 8:**
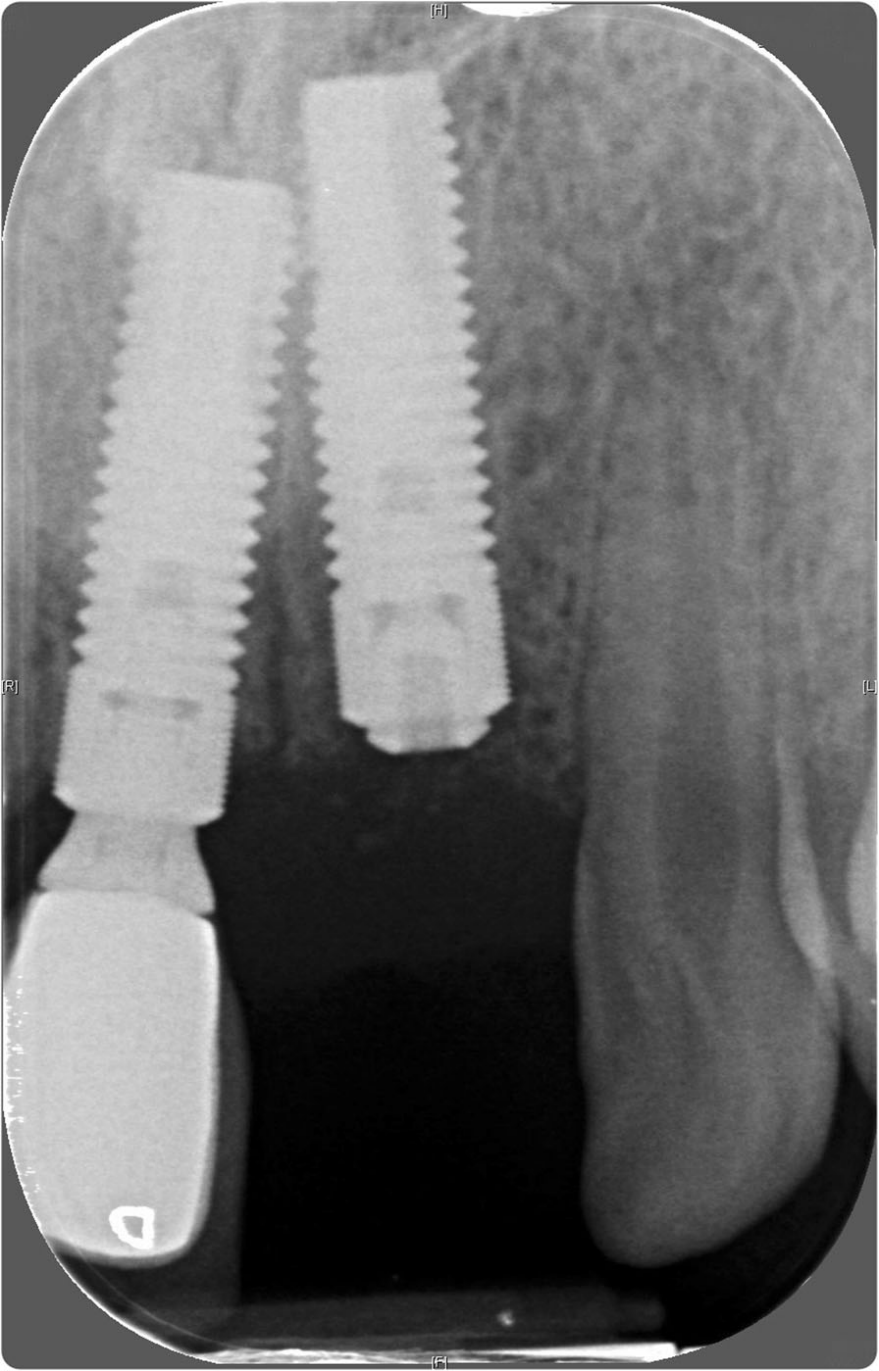
Placement confirmation. Radiograph of fixture in 21 position confirming angulation which is not ideal but acceptable

In July 2009, the patient presented with a draining sinus adjacent to 21 which could not be resolved with debridement and dressing of the root canal system and so was extracted, and the patient again was given a partial acrylic prosthesis. Ten months later, a 4.0-mm-diameter, 13-mm-length fixture (Astra OsseoSpeed©, Dentsply Implants, Mölndal, Sweden) was placed with simultaneous particulate xenograft (Geistlich BioOss©, Geistlich, Wolhusen, Switzerland) (Figs. 7 and 8). Following 5 months of osseointegration, the fixture was exposed and, in February 2012, restored with a cement-retained PFM restoration.

**Fig. 9:**
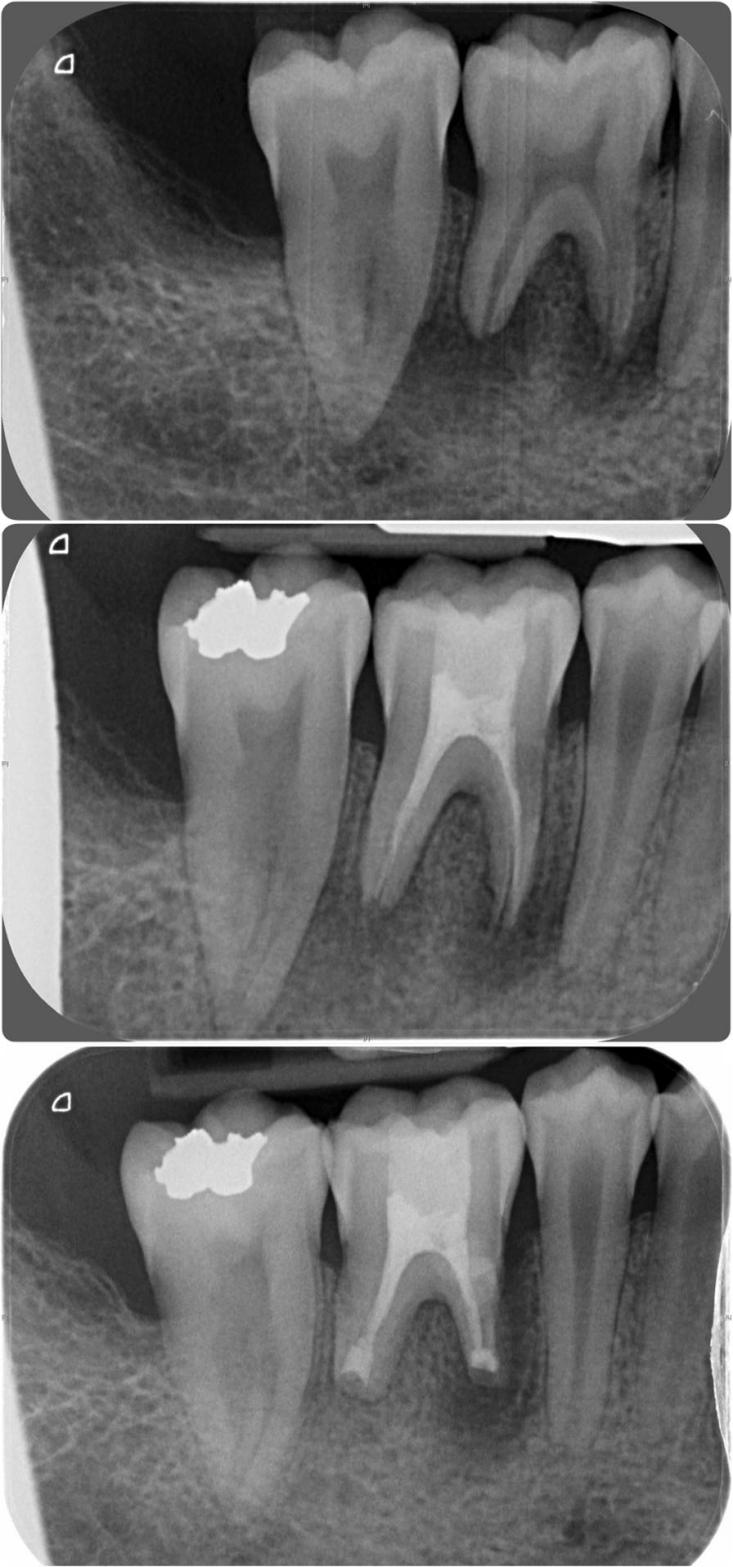
Failed endodontic treatments. Above, resorption associated with 46 and 47 and a periapical radiolucency associated with 46; middle, attempted root canal therapy of tooth 46; below, attempted intentional reimplantation with extra-oral root end surgery and retrograde root filling of tooth 46

**Fig. 10:**
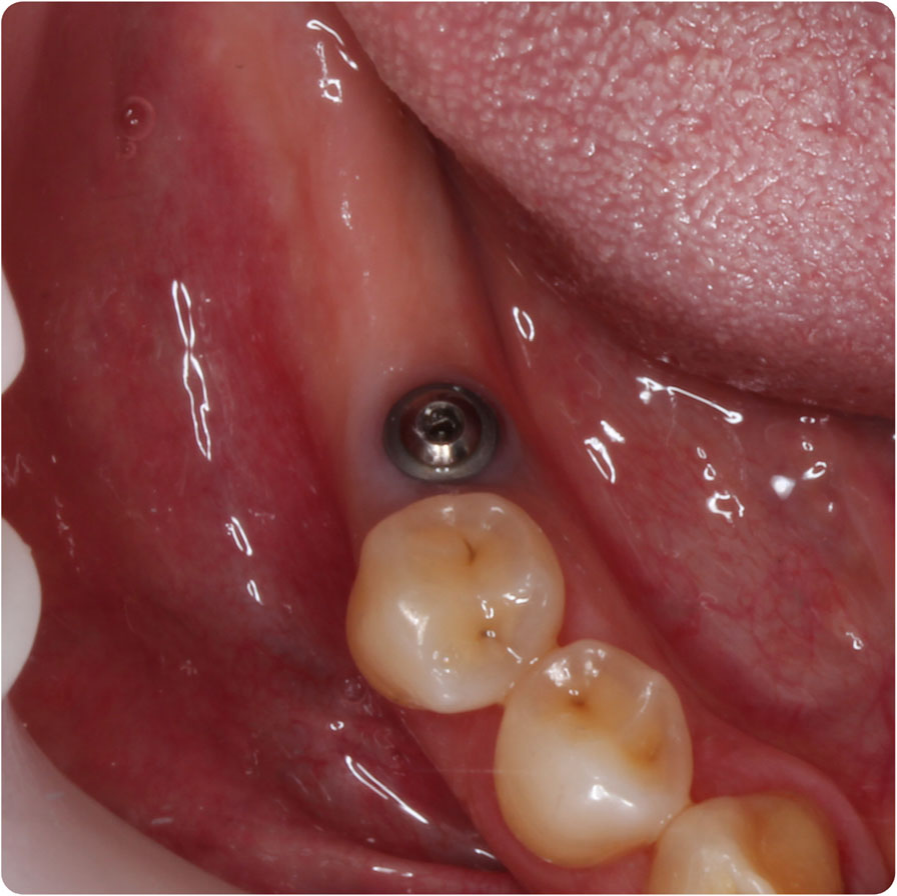
Most recent fixture photograph. Fixture in 46 position visible through the mucosa due to superficial positioning

**Fig. 11:**
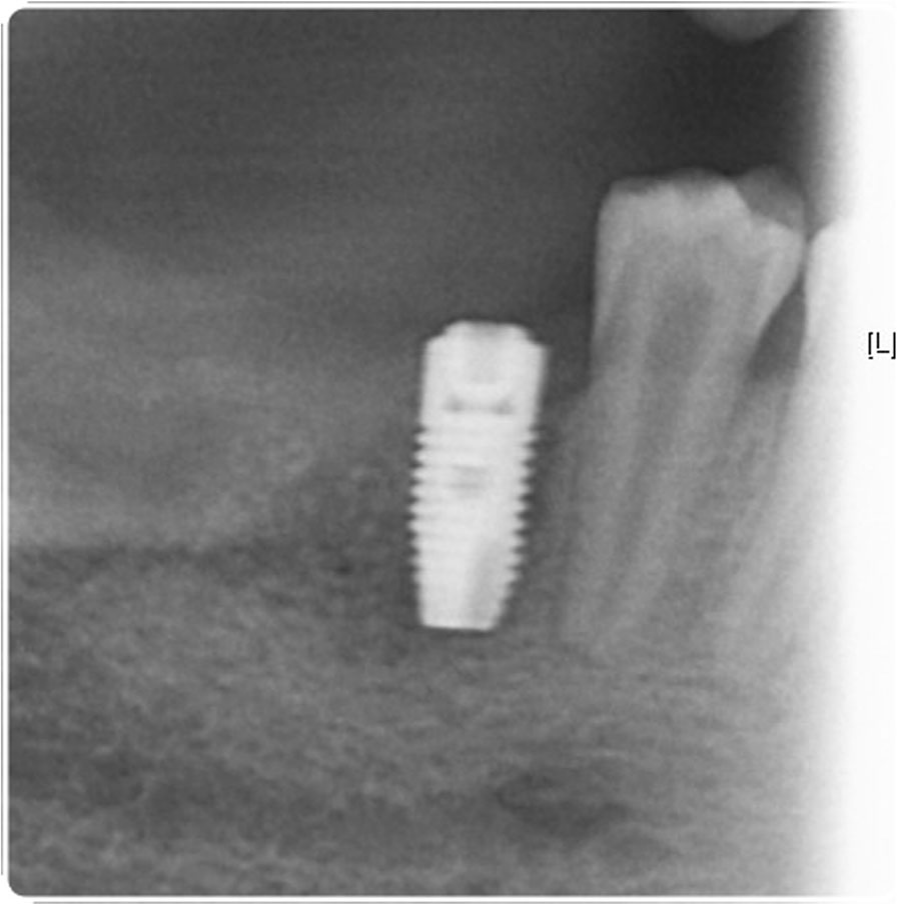
Most recent fixture radiograph. Radiograph of fixture in 46 position

In February 2014, the patient represented with a draining buccal sinus adjacent to 46 and 47, and plain film radiography and a CBCT scan showed root resorption of both teeth. Root canal therapy of 46 was unsuccessful, as was an attempted intentional reimplantation (Fig. 9), and therefore, 46 was removed 2 months later and 47 removed 15 months later. In May 2016, a 4.0-mm-diameter, 11-mm-length fixture (Astra OsseoSpeed©, Dentsply Implants, Mölndal, Sweden) was placed into the 46 position and was planned for exposure 4 months later, but due to the superficial position of the fixture, the cover screw became exposed spontaneously (Figs. 10 and 11). The fixture was restored with a screw-retained PFM restoration in January 2018.

**Fig. 12:**
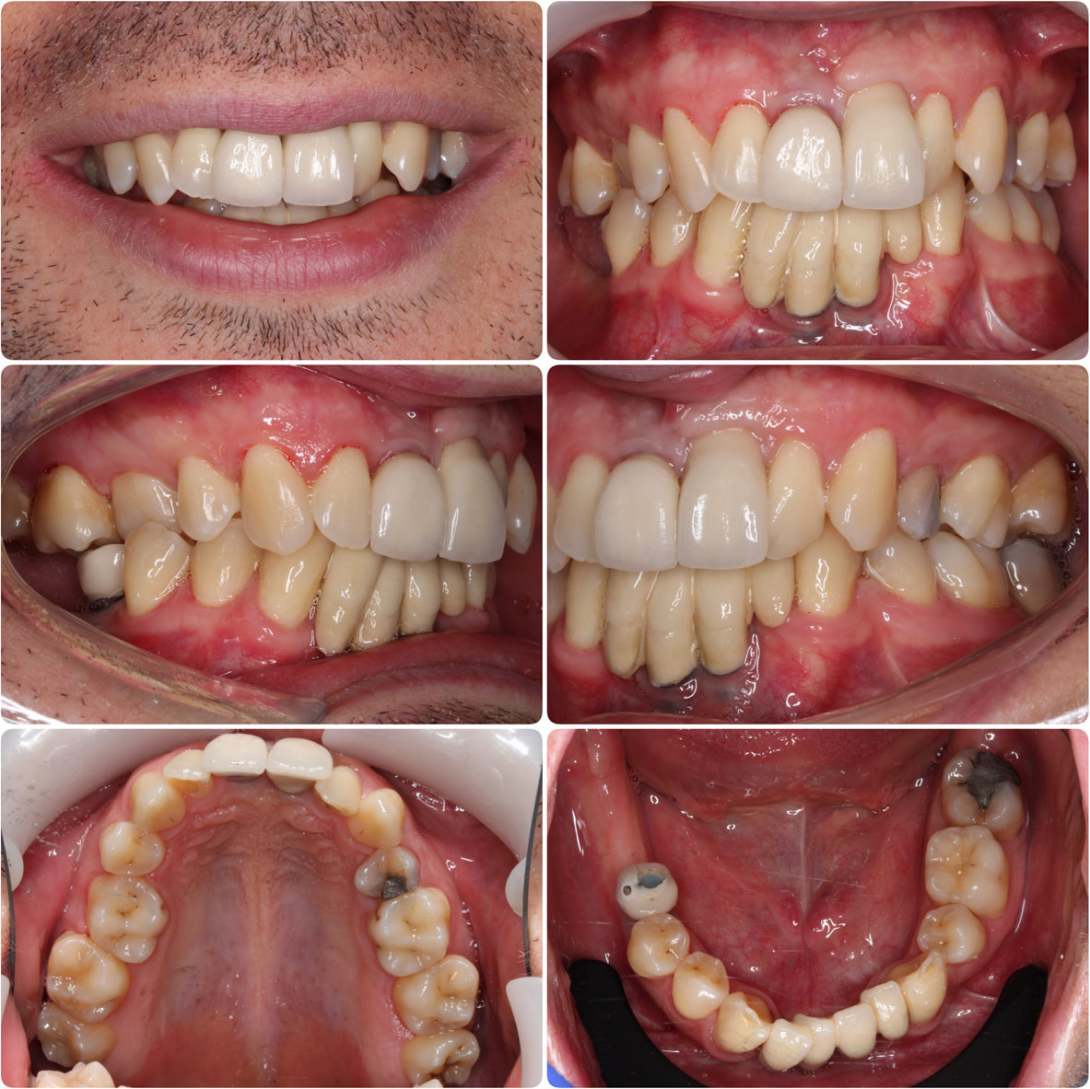
Follow-up photographs. 11, 31 and 41 position fixtures 123 months, 21 position fixture 80 months and 46 position fixture 21 months since placement

**Fig. 13:**
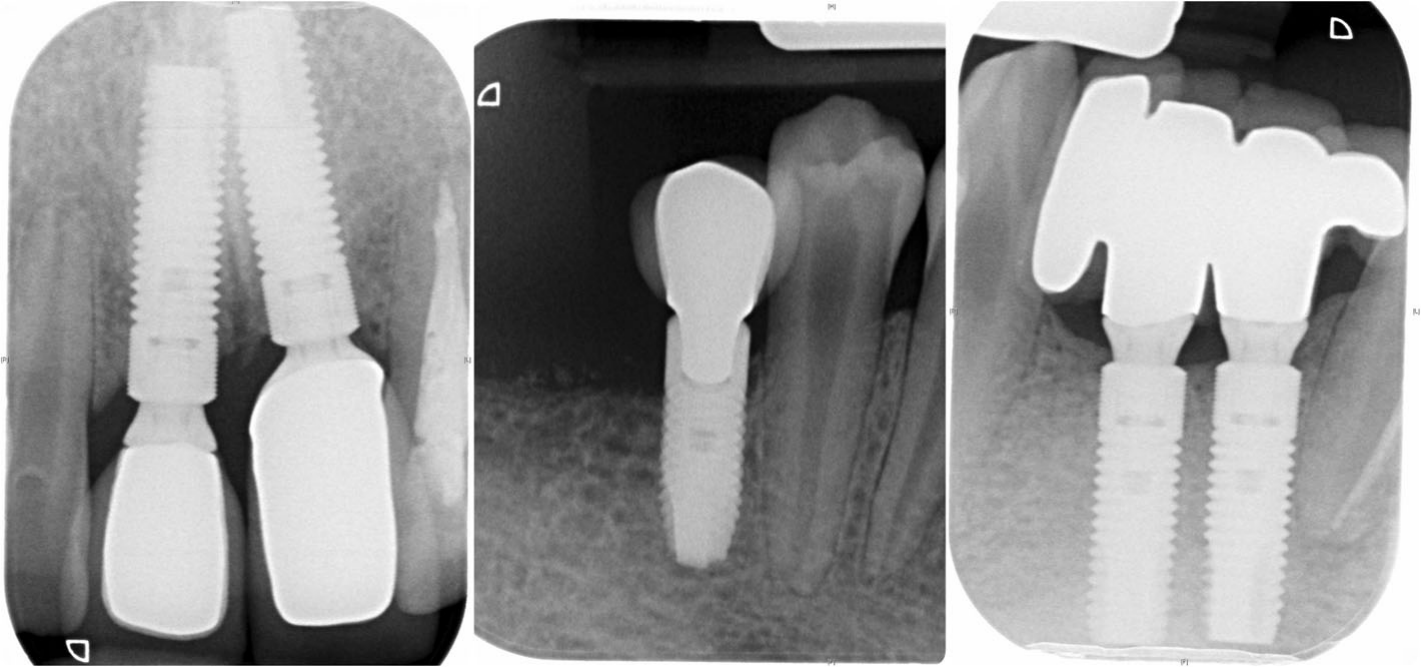
Follow-up radiographs. 11, 31 and 41 position fixtures 123 months, 21 position fixture 80 months and 46 position fixture 21 months since placement

At most recent follow-up (11, 31 and 41 position fixtures 123 months, 21 position fixture 80 months and 46 position fixture 21 months since placement), there was healthy, non-inflamed peri-implant mucosa (Fig. 12), no complications and no significant bone loss compared with baseline (Fig. 13).

## Discussion

Previous literature on the use of dental implants in patients with XLH is restricted to only a few case reports. Resnick [40] described a single implant placement, along with a particulate bone augmentation, with no complications at 40 months post insertion. Friberg [41] reported three case reports; one having four implants followed up for 31–50 months with a complication of rotational instability at the initial exposure of two implants (this resolved following an additional 8-month osseointegration period), another having xenograft augmentation prior to placement of four implants with no complications at 20 months, and the third case undergoing edentulous reconstruction with six maxillary and four mandibular implants supporting full arch fixed restorations with no complications at 19–22 months. Bergendal and Ljunggren [42] showed less success in their collection of three cases where a total of ten implants had been placed with eight of those lost, and Lee et al. [43] reported a poor outcome with the placement of two implants which remained hypermobile at 18 months’ post insertion and were not restored.

In the above case, a connective tissue graft was utilised to increase the width of the keratinised tissue. It was deemed necessary due to the patient reporting pain on brushing the healing abutments and presenting with poor oral hygiene around them. There are no previous papers specific on periodontal plastic surgery in patients with XLH for either teeth or dental implants. In general, the need for keratinised mucosa around dental implants is a contentious issue with different findings in the literature as to its effect on bone levels and implant survival. There is a reasonable volume of evidence to support the view that a lack of keratinised mucosa may lead to tooth brushing discomfort and therefore plaque accumulation, and for that reason, periodontal plastic surgery to increase the band of keratinised mucosa should be reserved for those patients who complain of brushing discomfort and in whom oral hygiene measures are impaired [44, 45].

This case has a longer follow-up of implant-supported restorations in a patient with XLH than any available in the published literature. The case emphasises that with thorough patient selection, planning, execution and maintenance, a reconstruction with implant-supported prostheses can be successful in the long term in this patient group who are more likely to require replacement of multiple missing teeth at a relatively young age than the general population.

## Abbreviations

11: Upper right central incisor
21: Upper left central incisor
31: Lower left central incisor
32: Lower left lateral incisor
41: Lower right central incisor
42: Lower right lateral incisor
46: Lower right first molar
47: Lower right second molar
ADJ: Amelo-dentinal junction
PFM: Porcelain-fused-to-metal
XLH: X-linked hypophosphatemia
